# A Systematic Review of Methods and Study Quality of Economic Evaluations for the Treatment of Schizophrenia

**DOI:** 10.3389/fpubh.2021.689123

**Published:** 2021-10-20

**Authors:** Luying Wang, Fenghao Shi, Xin Guan, He Xu, Jing Liu, Hongchao Li

**Affiliations:** ^1^School of International Pharmaceutical Business, China Pharmaceutical University, Nanjing, China; ^2^Center for Pharmacoeconomics and Outcomes Research, China Pharmaceutical University, Nanjing, China; ^3^Sumitomo Pharma (Suzhou) Co., Ltd., Shanghai, China

**Keywords:** schizophrenia, economic evaluation, systematic review, modeling, method review

## Abstract

**Background:** Schizophrenia is a severe and complex disease with substantial economic and social burdens. Despite multiple treatment choices, adverse events, and impaired social functions are still challenges in clinical therapy. Pharmacoeconomic evaluations could provide evidence to help decision makers improve the utilization of scarce resources. However, there remains some challenges especially in modeling due to uncertainties in progression of schizophrenia. There are limited summaries about the overall methodologies of schizophrenia economic evaluations.

**Objective:** The aim of this study is to review the existing economic evaluations of antipsychotics for the treatment of schizophrenia and summarize the evidence and methods applied.

**Methods:** An electronic literature search was performed in PubMed, Web of Science, EBSCO host, The Cochrane Library and ScienceDirect from January 2014 to December 2020. Search terms included “schizophrenia,” “schizophrenic,” “pharmacoeconomic,” “economic evaluation,” “cost-effectiveness,” and “cost-utility.” The Literature was screened and extracted by two researchers independently and assessed with the Quality of Health Economic Studies (QHES) List and Consolidated Health Economic Evaluation Reporting Standards (CHEERS) Statement.

**Results:** A total of 25 studies were included in the review. The regions included Europe, North America, Asia and Africa. Most of the studies chose second-generation antipsychotics as comparators and integrated treatment sequences. Time horizons varied from 1 year to lifetime. The healthcare sector was the most common perspective, accordingly, most of the evaluations considered only direct medical costs. The Markov model and decision tree model were the most common choices. Adverse events, compliance and persistence were considered important parameters. Quality-adjusted life-years were the major outcomes applied to the economic evaluations. All utilities for health states and adverse events were collected from published literature. All of the studies applied uncertainty analysis to explore the robustness of the results. The quality of the studies was generally satisfactory. However, improvements were needed in the choice of time horizons, the measurements of outcomes and the descriptions of assumptions.

**Conclusions:** This study highlights the methodology of economic evaluation of schizophrenia. Recommendations for modeling method and future study are provided.

## Introduction

Schizophrenia is a severe and complex mental illness with early onset coupled with behavior or cognitive disorders that have a significant impact on patients' family and society. A systematic review reported that the global age-standardized prevalence of schizophrenia was 0.28% and the prevalence of cases rose from 13.1 million cases in 1990 to 20.9 million cases in 2016 (1). The average annual healthcare costs were estimated to be between $23,887 and $24,988 according to a real-world retrospective study in the US (2). Patients may incur higher expenditures due to comorbidities that are common among them (3). Furthermore, indirect medical costs related to productivity lost or caregiving were 8.5 times higher compared with direct medical costs according to a retrospective study based on medical insurance database in Guangzhou, China (4). Also, schizophrenia has significant impact on caregivers of the patients. The well-being of both patients and caregivers could be affected during their cognitive appraisal processes of the illness, help-seeking experience and the interaction within the families (5) and the burden of caregivers exists in physical and mental health, social relationship, and financial life (6).

Economic evaluations could generate evidence incorporating both costs and consequences for decision makers to clarify different uses for scarce resources (7). Despite the multiple choices of medications in schizophrenia treatment, there still exist substantial burdens and difficulties in clinical therapies due to low adherence and adverse events. Thus, pharmacoeconomic evidence is required to balance the clinical effects with the resources consumed. However, there remain some challenges especially in modeling due to uncertainties in the progression of the diseases, emphasizing the requirements for systematic reviews of the methods applied in the analysis.

Previous systematic reviews have evaluated studies published since 2000 (8–10). However, none of them fully discussed the treatment sequences or methods applied. Furthermore, most of them adopted an extensive range of years of publication, which may not characterize the studies in recent years. Therefore, this study was conducted to review the model-based economic evaluations published recently for antipsychotics and summarize the modeling techniques, including model structures, basic settings, integration and translation of the clinical events, and selection of utility values. In addition, the review also aimed to assess the quality of the studies.

## Methods

The systematic review was conducted according to the Preferred Reporting Items for Systematic Reviews and Meta-analyses (PRISMA) statement developed by Moher et al. (11).

### Eligibility Criteria

The inclusion criteria were as follows: (a) economic evaluations adopting cost-effectiveness analysis (CEA) or cost-utility analysis (CUA) approach; (b) patients diagnosed with schizophrenia with no limitation on gender or age; (c) intervention including all antipsychotics; and (d) outcomes presented as incremental cost-effectiveness (ICER). Studies were excluded if they met the any of the following criteria: (a) not reported in English; (b) not related to economic evaluation; (c) cost of illness, health-related quality of life or budget impact analysis studies; (d) abstracts or studies with full-text unavailable; (e) not model-based studies; and (f) chose clinical effect as the only outcome.

### Search Strategy

An electronic literature search was performed in PubMed, Web of Science, EBSCO host, The Cochrane Library, ScienceDirect from January 2014 to December 2020. Search items included “schizophrenia,” “schizophrenic,” “pharmacoeconomics,” “economic evaluation,” “cost-effectiveness,” and “cost-utility.” The detailed strategy is provided in Supplementary Material. In addition, references from retrieved studies were searched manually to avoid missing data.

### Data Extraction and Analysis

The included studies were screened, extracted and double checked by two researchers independently. Disagreements were resolved by discussion or by consulting a third researcher. General information was collected including title, first author's surname, year of publication, country or region, intervention, and treatment sequences. To summarize the methods applied, characteristics such as perspectives, type of costs, outcomes, model structures, and necessary parameters were recorded. The results and conclusions of studies were included in the extracted form but were not reported as main outcomes due to the arguments regarding the extrapolation of evaluation results (12). All the information was recorded and compared using Microsoft Excel 2016.

### Quality Assessment

According to the review of quality assessment tools conducted by Walker et al. (13) in 2012, the Quality of Health Economic Studies (QHES) List was recommended to discriminate the quality of studies as a quantitative measurement. The Consolidated Health Economic Evaluation Reporting Standards (CHEERS) Statement (14) published in 2012 was recommended by the International Society for Pharmacoeconomics and Outcomes research (ISPOR) as a report checklist and for guidance to optimize the quality of reporting. Both QHES and CHEERS were applied to quantitatively and qualitatively assess the quality of studies.

## Results

**Figure 1:**
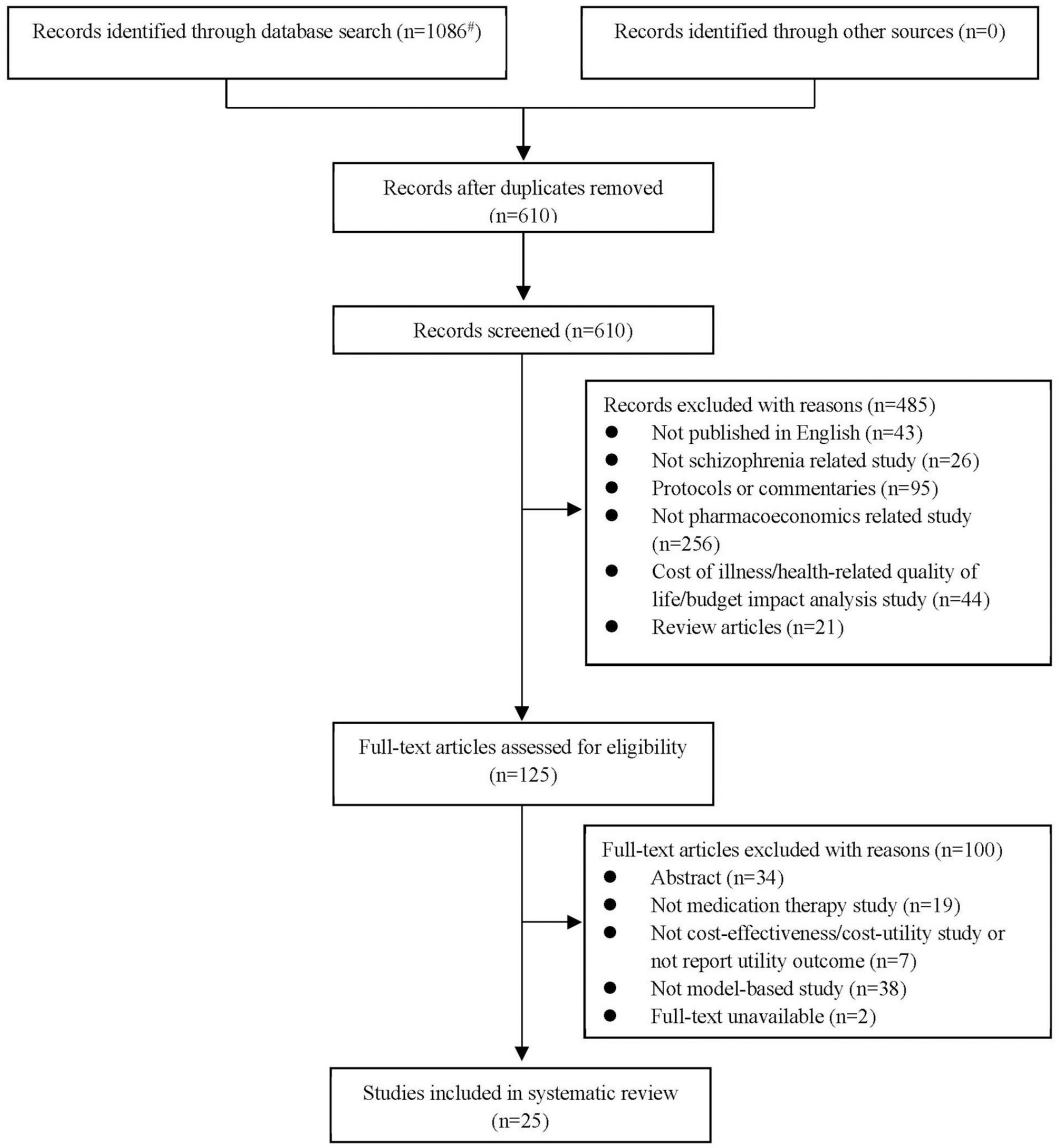
Flow of information through different phases of the systematic review according to the PRISMA statement [^#^Detailed search results: PubMed Database (*n* = 202), Web of Science Database (*n* = 377), EBSCO host Database (*n* = 113), The Cochrane Library Database (*n* = 286), ScienceDirect Database (*n* = 108)].

A total of 1,086 citations were retrieved from five electronic databases. After removing duplicates, 610 studies were eligible to enter the screening process and judgements were generated according to the titles and abstracts. Finally, 25 articles published in English were identified and included in the systematic review. A flow of the literature screening was provided in Figure 1 according to the PRISMA statement (11).

### Basic Characteristics of Included Studies

The characteristics of the included studies were summarized in Table 1. The studies covered the regions of Europe (15, 18, 21, 23–28, 32, 34–36, 39), North America (16, 17, 31), Asia (19, 22, 29, 30, 33, 37, 38), and Africa (20). Eleven (44%) (16, 17, 19–22, 29, 32, 36, 37, 39) of the 25 studies specified the onset or averaged ages of patients and four studies (16%) (15, 18, 23, 24) focused on patients in acute or relapsed states. Second-generation antipsychotics (SGAs) also known as atypical antipsychotics were the most common interventions chosen in the analyses, where seven studies (28%) (15, 18, 21, 24, 25, 27, 30) compared the cost-effectiveness of long-acting-injections (LAIs). Eight studies (32%) (18–22, 25, 28, 39) included both first-generation and second-generation antipsychotics as compared interventions where haloperidol was the commonest (*n* = 7, 87.5%). Most of the studies measured the outcomes using quality-adjusted life-years (QALYs) (*n* = 23, 92%), while only 2 studies (8%) (19, 20) applied disability-adjusted life-years (DALYs). In addition, nine studies (18, 21, 24, 25, 27, 28, 30, 31, 33) reported both utility and clinical effects, where yearly relapse or relapse avoidance was the most frequent choice.

**Table 1:** Basic characteristics.

**References**	**Country/region**	**Patients**	**Interventions**	**Perspectives**	**Type of Costs**	**Outcome measurement**	**Discount rate**
Einarson et al. (15)	Sweden	Schizophrenia patients with relapse	Paliperidone LAI, olanzapine LAI, risperidone LAI, haloperidol LAI, oral olanzapine	Health care sector perspective	Direct and indirect costs	QALYs	NA
Lachaine et al. (16)	Canada	Moderate to severe schizophrenia patients above 40	Asenapine, olanzapine	Health care sector perspective, societal perspective	Direct and indirect costs	QALYs	5%
Park et al. (17)	United States	Schizophrenia patients above 40	Olanzapine, risperidone, quetiapine, ziprasidone	Health care sector perspective	Direct costs	QALYs	3%
Dilla et al. (18)	Spain	Schizophrenia patients in relapse due to low compliance	Olanzapine LAI, risperidone LAI	Health care sector perspective	Direct costs	QALYs, relapse averted, life years	3%
Anh et al. (19)	Vietnam	Schizophrenia patients above 15	Chlorpromazine, haloperidol, levopromazine, risperidone, clozapine, olanzapine	Health care sector perspective	Direct costs	DALYs	3%
Lubinga et al. (20)	Uganda	Schizophrenia patients with average age of 25	Chlorpromazine, haloperidol, risperidone, olanzapine, quetiapine	Societal perspective	Direct and indirect costs	DALYs	3%
Druais et al. (21)	France	Stable schizophrenia patients with average age of 38	Paliperidone LAI, risperidone LAI, aripiprazole LAI, olanzapine LAI, haloperidol LAI, oral olanzapine	Payer perspective	Direct costs	QALYs, relapse averted	4%
Lin et al. (22)	Singapore	Schizophrenia patients with average age of 37	Amisulpride, aripiprazole, chlorpromazine, olanzapine, paliperidone, quetiapine, risperidone, sulpiride, trifluoperazine, ziprasidone	Health care sector perspective	Direct costs	QALYs	3%
Rajagopalan et al. (23)	Scotland and Wales	Schizophrenia patients in relapse	Lurasidone, aripiprazole	Payer perspective	Direct costs	QALYs	3.50%
Einarson et al. (24)	Finland	Schizophrenia patients in relapse	Aripiprazole LAI, paliperidone LAI, olanzapine LAI, risperidone LAI	Health care sector perspective	Direct costs	QALYs, relapse averted	NA
Einarson et al. (25)	Portugal	Schizophrenia patients	Paliperidone LAI, risperidone LAI, haloperidol LAI, oral olanzapine	Health care sector perspective	Direct costs	QALYs, relapse averted	NA
Barnes et al. (26)	United Kingdom	Patients unresponsive to clozapine	Olanzapine, amisulpride	Societal perspective	Direct costs	QALYs	Not specified
Einarson et al. (27)	Spain	Schizophrenia patients	PP3M, PP1M	Health care sector perspective	Direct costs	QALYs, relapse averted, hospitalization averted	NA
Einarson et al. (28)	Netherlands	Schizophrenia patients	PP3M, PP1M, haloperidol LAI, risperidone microspheres, oral olanzapine	Payer perspective	Direct costs	QALYs, relapse (hospitalization treated or out-patient treated)	NA
Wiwat et al. (29)	Thailand	Stable schizophrenia patients above 15	Aripiprazole, risperidone	Societal perspective	Direct costs	QALYs	3%
Nuhoho et al. (30)	United Arab Emirates	Stable schizophrenia patients	Paliperidone LAI, other oral antipsychotics	Payer perspective	Direct costs	QALYs, rate of hospitalization, relapse, emergency	NA
Aigbogun et al. (31)	United States	Stable schizophrenia patients	Brexpiprazole, cariprazine, lurasidone	Payer perspective	Direct costs	QALYs, relapse averted, hospitalization averted	NA
Németh et al. (32)	Hungary	Patients with negative symptoms of schizophrenia with average age of 40	Cariprazine, risperidone	Payer perspective	Direct costs	QALYs	4%
Zhao et al. (33)	China	Schizophrenia patients	Olanzapine ODT, olanzapine SOT, aripiprazole SOT	Payer perspective	Direct costs	QALYs, averaged annual relapse	NA
Abdall-Razak et al. (34)	United Kingdom	Not specified	Paliperidone, amisulpride	Payer perspective	Direct costs	QALYs	NA
Dutina et al. (35)	Serbian	Adult patients about to receive for the second-line treatment	Aripiprazole, olanzapine	Payer perspective	Direct costs	QALYs	3%
Arteaga et al. (36)	France	Adult chronic schizophrenic patients stabilized on PP1M with baseline age of 38.75	PP3M, PP1M	Payer perspective	Direct costs	QALYs	4%
Yi et al. (37)	China	Schizophrenia patients with starting age of 35	Amisulpride, olanzapine	Payer perspective	Direct costs	QALYs	3%
Lin et al. (38)	China	Not specify	Aripiprazole ODT, aripiprazole SOT, olanzapine SOT	Payer perspective	Direct costs	QALY	NA
Jin et al. (39)	United Kingdom	Individuals referred to secondary care mental health services with mean age of 23.5	Amisulpride, aripiprazole, haloperidol, olanzapine, quetiapine, risperidone, placebo, clozapine	Payer perspective	Direct costs	QALYs	3.5%

*LAI, long-acting-injection; ODT, orally disintegrating antipsychotic tablets; SOT, standard oral tablets; PP3M, paliperidone administered every 3 months; PP1M, paliperidone administered every month; QALY, quality-adjusted life year; DALY, disability-adjusted life year; NA, not applicable*.

Due to the diverse efficacy and low compliance rate, therapeutic changes are common among schizophrenia patients, which makes treatment sequences worth consideration. Nineteen studies (76%) (15–18, 20–25, 27–31, 33, 35, 36, 38) specified treatment sequences in the models (Table 2). The methods of setting second-line medications were flexible, including changing to drugs that differed from the first-line or applying mixed therapies based on market share (22, 30, 36) or simple averaging (22, 31). Clozapine was the most common last-line therapy.

**Table 2:** Treatment sequences^#^ in the included studies.

**References**	**Consideration of the treatment sequence**	**First-line treatment**	**Second-line treatment**	**Third-line treatment**
Einarson et al. (15)	Yes	Paliperidone LAI or olanzapine LAI or risperidone LAI or oral olanzapine or haloperidol LAI	Olanzapine LAI or paliperidone LAI or haloperidol LAI or oral olanzapine^*^	Clozapine
Lachaine et al. (16)	Yes	Asenapine or olanzapine	Aripiprazole or ziprazidone or risperidone or quetiapine	NA
Park et al. (17)	Yes	Olanzapine or risperidone or quetiapine or ziprasidone	Olanzapine or risperidone or quetiapine or ziprazidone^*^	Clozapine
Dilla et al. (18)	Yes	Olanzapine LAI or risperidone LAI	Other antipsychotics	NA
Anh et al. (19)	No	Chlorpromazine or haloperidol or levopromazine or risperidone or clozapine or olanzapine	NA	NA
Lubinga et al. (20)	Yes	Chlorpromazine or haloperidol or risperidone or olanzapine or quetiapine	Risperidone or haloperidol^*^	Perphenazine
Druais et al. (21)	Yes	Paliperidone LAI or risperidone LAI or aripiprazole LAI or olanzapine LAI or haloperidol LAI or oral olanzapine	25%paliperidoneLAI+25%risperidone LAI+25%aripiprazoleLAI+25%olanzapine LAI^**^	NA
Lin et al. (22)	Yes	Amisulpride or aripiprazole or chlorpromazine or haloperidol or olanzapine or paliperidone or quetiapine or risperidone or sulpiride or trifluoperazineor ziprazidone	The other drugs excluded the first-line drugs^***^	Clozapine
Rajagopalan et al. (23)	Yes	Lurasidone or aripiprazole	Amisulpride	Clozapine
Einarson et al. (24)	Yes	Aripiprazole or paliperidone or olanzapine or risperidone	Olanzapine or risperidone^*^	Clozapine
Einarson et al. (25)	Yes	Paliperidone or risperidone or haloperidol or olanzapine	Haloperidol or olanzapine^*^	Clozapine
Barnes et al. (26)	No	Olanzapine or amisulpride	NA	NA
Einarson et al. (27)	Yes	PP3M or PP1M	Aripiprazole	Clozapine
Einarson et al. (28)	Yes	PP3M or PP1M or haloperidol or risperidone or olanzapine	Haloperidol or oral olanzapine	Clozapine
Wiwat et al. (29)	Yes	Aripiprazole or risperidone	Clozapine	NA
Nuhoho et al. (30)	Yes	Paliperidone LAI or paliperidone LAI plus oral antipsychotics	Risperidone or paliperidone or aripiprazole or olanzapine or quetiapine^***^	Risperidone or paliperidone or aripiprazole or olanzapine or quetiapine^*^
Aigbogun et al. (31)	Yes	Brexpiprazole or cariprazine or lurasidone	Olanzapine or risperidone or quetiapine or ziprazidone or aripiprazole^**^	NA
Németh et al. (32)	No	Cariprazine or risperidone	NA	NA
Zhao et al. (33)	Yes	Olanzapine ODT or olanzapine SOT or aripiprazole SOT	Aripiprazole or amisulpride or ziprazidone or clozapine	NA
Abdall-Razak et al. (34)	No	Paliperidone or amisulpride	NANA	
Dutina et al. (35)	Yes	Aripiprazole or olanzapine	Clozapine	NA
Arteaga et al. (36)	Yes	PP3M or PP1M	Paliperidone, olanzapine, aripiprazole, risperidone^***^	NA
Yi et al. (37)	No	Amisulpride or olanzapine	NA	NA
Lin et al. (38)	Yes	Aripiprazole ODT or aripiprazole SOT or olanzapine SOT	Not specify	NA
Jin et al. (39)	No	Amisulpride, aripiprazole, haloperidol, olanzapine, quetiapine, risperidone, placebo, clozapine	NA	NA

*LAI, long-acting-injection; ODT, orally disintegrating antipsychotic tablets; SOT, standard oral tablets; PP3M, paliperidone administered every 3 months; PP1M, paliperidone administered every month; NA, not applicable*.

# *If patients show no response to the therapies or relapse during the current treatment, they will change to the next-line treatment*.

* *A single drug different from the previous treatment line was chosen as the current treatment line*.

** *Clinical and economic inputs were determined by a weighted average with equal proportions of data for the drugs*.

*** *Clinical and economic inputs were determined by market share of the drugs*.

### Results of Methodology Review

#### Perspectives and Related Costs

Various terms were used to define the study perspectives of the reviewed articles. Therefore, the terms were classified into three categories in this review, which were defined as health care sector perspective (including healthcare system, ministry of health, national health service, and government), payer perspective (including payer, third-party payer, and health insurance), and societal perspective (including societal, modified societal, and broadly societal perspective) based on the report recommended by ISPOR (40). Notably, studies of single-payer health system countries using both the healthcare system and payer were classified into the payer perspective. The perspectives determine the types of costs considered in the analysis (7, 40). Direct medical costs, both direct and indirect costs, and costs paid by payers are most relevant to the health care sector perspective, the societal perspective, and the payer perspective, respectively (7, 41).

Among the eight studies (32%) choosing the health care sector perspective (15, 17–19, 22, 24, 25, 27), seven studies adapted direct medical costs, while one study (15) included indirect costs which is inconsistent with the perspective. The payer perspective was chosen by 13 studies (52%) (21, 23, 28, 30–39) where six studies specified costs paid by payers and seven studies (28, 31, 33, 35–38) merely included direct medical costs. Among the 3 (14.3%) societal perspective-studies (20, 26, 29), only 1 study (20) took both direct and indirect costs into account, indicating that some confusion existed in distinguishing perspectives. One study chose both the healthcare sector and the societal perspective with direct and indirect costs (16).

#### Types of Models and Health States

The characteristics of the models and related health states are summarized in Table 3. The Markov model (17, 19, 20, 22, 23, 26, 29, 32, 35–37) and the decision tree model (15, 24, 25, 27, 28, 30, 34) were the most common choices. One study (16) combined the decision tree model with the Markov model to better reflect complication-related treatment switches within the first year of treatment and long-term metabolic complications. Most of the Markov models consisted of a series of health states, which represent treatment sequences, disease progression or related adverse events and were connected by probabilities based on the averaged cohort level (27). Microsimulation models (18, 33, 38, 39) are more flexible and natural for simulating clinical reality by incorporating patient-level characteristics. Due to the differentiation in the choice of therapy and the treatment effect among patients, a patient-level simulation model as well as a Markov model incorporating treatment sequences, relapse, remission, adherence and adverse events could be more suitable for clinical practice.

**Table 3:** Basic characteristics of the models in the included economic evaluations.

**References**	**Types of model**	**Model states**	**Time horizon**	**Cycle length**
Einarson et al. (15)	Decision tree, cohort	Incorporating clinical events including discontinuation, exacerbation, compliance, hospitalization	1 year	NA
Lachaine et al. (16)	Decision tree combined with 9-state Markov model, cohort	Diabetes, stroke, CHDs, hypertension, no comorbidity, 2/3/4 comorbidities, death	5–10 years	1 year
Park et al. (17)	9-state Markov model, cohort	First line treatment with/without irreversible SE, 2nd line treatment with/without irreversible SE, clozapine treatment with/without irreversible SE, uncontrolled state with/without SE, death	10 years	18 weeks
Dilla et al. (18)	Discrete event simulation, microsimulation	Treatment, treatment emergent adverse events, relapse, doctor-initiated treatment re-evaluation, patient-initiated treatment discontinuation	5 years	NA
Anh et al. (19)	3-state Markov model, cohort	Schizophrenia patients, recovery patients, schizophrenia-specific and other causes of deaths	Lifetime	1 year
Lubinga et al. (20)	10-state Markov model, cohort	Residual on/off 1st line AP, acute on/off 1st line AP, residual on/off 2nd line AP, acute on/off 2nd line AP, residual on 3rd line	Lifetime	1 year
Druais et al. (21)	4-state Markov model, cohort	Stable treated, stable non-treated, relapse, death	5 years	3 months
Lin et al. (22)	4-state Markov model, cohort	Stable treated, stable non-treated, relapse, death	Lifetime	1 year
Rajagopalan et al. (23)	5-state Markov model, cohort	Non-stable/relapse trial of antipsychotic agents, stable/adherent, stable/non-adherent, relapse, death	10 years	6 weeks
Einarson et al. (24)	decision tree, cohort	Incorporating clinical events including discontinuation, exacerbation, compliance, hospitalization	1 year	NA
Einarson et al. (25)	decision tree, cohort	Incorporating clinical events including discontinuation, exacerbation, compliance, hospitalization	1 year	NA
Barnes et al. (26)	3-state Markov model, cohort	Symptom response, SEs, death	1–10 years	3 months
Einarson et al. (27)	decision tree, cohort	Incorporating clinical events including discontinuation, exacerbation, compliance, hospitalization	1 year	NA
Einarson et al. (28)	decision tree, cohort	Incorporating clinical events including stable, intolerant, relapse treated as out-patient, relapse requiring hospitalization and dropout	1 year	NA
Wiwat et al. (29)	4-state Markov model, cohort model	Remission with 1st antipsychotics, relapse, remission with clozapine, death	Lifetime	4 weeks
Nuhoho et al. (30)	Decision tree, cohort	Incorporating clinical events including adherence, exacerbation, hospitalization	1 year	3 months
Aigbogun et al. (31)	Decision-analytic model, cohort	Incorporating clinical events including treatment discontinuation, relapse/impending relapse, AEs	1 year	NA
Németh et al. (32)	8-state Markov model, cohort	Constructed according to both severity of symptoms and disease types	1–10 years	1/12 weeks
Zhao et al. (33)	Decision-analytic model, microsimulation	Incorporating adherence levels, relapse with/without hospitalization, treatment discontinuation, AEs suicide risk	1 year	3 months
Abdall-Razak et al. (34)	Decision tree, cohort	Incorporating relapse, remission, AEs, diabetes complications	1 year	NA
Dutina et al. (35)	5-state Markov model, cohort	Remission without AEs, remission with AEs, relapse, second response, death	10 years	3 months
Arteaga et al. (36)	5-state Markov model, cohort	1st-line treatment, no active treatment, 2nd-line treatment, relapse, death	5 years	1 month
Yi et al. (37)	5-state Markov model, cohort	Acute phase, remission, relapse, death	Lifetime	1 year
Lin et al. (38)	Discrete event simulation, microsimulation	Incorporating adherence levels, relapse with/without hospitalization, stable and adverse events	1 year	NA
Jin et al. (39)	Discrete event simulation, microsimulation	Incorporating 4 module for different pathway with relevant interventions	Lifetime	NA

*CHDs, coronary heart diseases; AP, antipsychotics; SEs, side effects; AEs, adverse events; NA, not applicable*.

#### Time Horizon and Cycle Length

As summarized in Tables 3, **6** (24%) studies (19, 20, 22, 29, 37, 39) chose the lifetime horizon in the model. However, considering the low adherence and frequent changes of medication, a certain number of studies (15, 16, 18, 21, 24–27, 30–33, 35) chose a relatively short time horizon. Among these, most Markov model-based studies explored uncertainty by extending the time horizon as a complementary analysis, while most decision tree model-based or microsimulation model-based studies conducted mere 1-year analyses (15, 24, 25, 27, 30, 31, 33, 34). As a chronic mental disease, schizophrenia should be simulated for a long time period or even lifetime in the model. However, due to the uncertainty in therapy and disease progression, it might be challenging to simulate further into the future as it becomes more unpredictable. Therefore, selecting the appropriate time frame covering events in the near future and then exploring time horizon uncertainty may be a reasonable method for economic evaluations for schizophrenia.

The cycle lengths of Markov models in the included studies varied from 4 weeks to 1 year, where 3 months (21, 26, 30, 32, 33, 35) and 1 year (16, 19, 20, 22, 37) were the most frequently used. The reasons for 3-month cycle selection included appropriate capture the both clinical practice and associated events according to clinical opinion (21, 30), consistent with clinical trials (26), while explanation for 1-year cycle selection was consideration of the realistic treatment management of schizophrenia (37). The length of cycle selection should be depend on both disease and intervention (7), and should be short enough to avoid multiple changes within a single cycle (42). Therefore, a 1-year cycle length may be less preferable compared to a 3-month cycle length.

#### Adverse Events

Adverse events (AEs) could impact adherence, efficacy and therapy changes as well as health-related quality of life, especially for schizophrenia. Thus, it is meaningful to take relevant AEs into account in the models. The AEs selected in the evaluations are listed in Table 4. The effect of the extrapyramidal system (EPS), weight gain, and diabetes were the most common adverse events considered. A few studies built separate health states for possible AEs to reflect the influence by applying transition probabilities, costs and utility of the health states (16, 22, 26). The majority of studies incorporated the prevalence, costs and disutility of AEs in each cycle to reflect the impact on disease progression. Both methods were acceptable as long as the choice was made based on proper consideration of the disease progression and available evidence (42).

**Table 4:** Summary of the adverse events considered in the included economic evaluations.

**References**	**Consideration and description of AEs**	**Types of AEs**					
		EPS^*^	**Weight gain**	**Diabetes**	**Hyperprolactinemia**	Metabolic events^**^	**Others**
Einarson et al. (15)	×	×	×	×	×	×	NA
Lachaine et al. (16)	√	√	√	√	×	√	NA
Park et al. (17)	√	√	×	×	√	√	Agranulocytosis
Dilla et al. (18)	√	√	√	×	×	×	Somnolence, sexual dysfunction, postinjection syndrome, suicide
Anh et al. (19)	√	√	√	×	×	×	Agranulocytosis
Lubinga et al. (20)	√	√	√	√	×	×	Ischemic heart disease
Druais et al. (21)	√	√	√	√	×	×	NA
Lin et al. (22)	√	√	√	√	×	×	NA
Rajagopalan et al. (23)	√	√	√	√	×	×	NA
Einarson et al. (24)	×	×	×	×	×	×	NA
Einarson et al. (25)	×	×	×	×	×	×	NA
Barnes et al. (26)	√	√	√	×	×	√	Sexual dysfunction, aversive subjective experience, cardiac symptoms
Einarson et al. (27)	×	×	×	×	×	×	NA
Einarson et al. (28)	×	×	×	×	×	×	NA
Wiwat et al. (29)	√	√	√	√	√	×	NA
Nuhoho et al. (30)	×	×	×	×	×	×	NA
Aigbogun et al. (31)	√	√	√	×	×	√	NA
Németh et al. (32)	√	√	×	×	×	×	NA
Zhao et al. (33)	√	√	√	√	×	√	NA
Abdall-Razak et al. (34)	√	√	√	√	×	×	Diabetes complications: amputation, MI, stroke, IHD, HF
Dutina et al. (35)	√	√	×	×	×	√	Neutropenia
Arteaga et al. (36)	√	√	√	√	√	×	NA
Yi et al. (37)	√	√	√	×	√	√	Liver function damage
Lin et al. (38)	√	√	√	√	√	√	NA
Jin et al. (39)	√	√	√	√		√	Neutropenia

*AEs, adverse events; MI, myocardial infarction; IHD, ischemic heart disease; HF, heart failure; NA, not applicable*.

* *EPS (effects of the extrapyramidal system) including akathisia, and tardive dyskinesia*.

** *Metabolic events including pathoglycemia, dyslipidemia and hypertension*.

However, some studies did not fully describe the consideration of AEs (27, 30) or even did not include AEs (15, 24, 25, 28) due to the similar incidence rates, small expenditures or short time horizon. It should be discussed whether the 1-year time horizon was sufficient to capture all important and interesting outcomes, since such a time horizon might fail to capture the impact on both health-related quality of life and costs of relevant clinical events among drugs. It is also notable that AEs with different durations should be clearly described to calculate the respective additional costs and disutilities. Park et al. (17) and Arteaga et al. (36) classified the reversible and irreversible AEs and assumed that reversible AEs would last for 18 weeks or 3 months while irreversible AEs would remain for the rest of the period. Rajagopalan et al. (23) classified the AEs into one-off AEs (such as EPS), persistent AEs (such as weight gain), and cumulatively occurred AEs (such as diabetes). Abdall-Razak et al. (34) clearly described the health-related utilization of the AEs and summarized the calculation of utilities of each AEs. However, more than half of the studies failed to clearly defined both costs and utilities considerations of the AEs.

#### Compliance and Persistence

Medication compliance (also known as adherence) can be defined as the extent to which the medication-taking of a patient matches that defined by the prescriber while medication persistence (also known as continuous adherence or discontinuation rate) refers to the act confirming to the recommended continuing treatment for the duration of time from the prescriber (43, 44). Both of them could influence the risk of relapse, rehospitalization, costs, quality of life through different aspects, especially for chronic disorders such as schizophrenia (44). Due to the different definitions and roles, it is important to not only take both compliance and persistence into account but also distinguish them when building economic models.

Information about the adoption of compliance or persistence in the included studies is listed in Table 5. The management of medication compliance or medication persistence can be summarized into three types: (1) Patients classified based on the compliance rates (adherence rates) from existing literature and then search for inputs under relevant compliance (15, 24, 25, 30, 33, 38). Such a method is usually applied in decision tree models and microsimulation models. (2) Non-persistence rates (discontinuation or dropout rates) are used rather than compliance to reflect the medication behaviors (17, 18, 20–22, 27, 28, 31, 36, 39). In such studies, non-persistence rates were divided into lack of efficacy, adverse events, intolerance, patients' choice and so on. Patients with treatment withdrawn due to lack of efficacy, adverse events or intolerance would change their therapies according to the treatment sequence. Patients who stopped treatment due to their own choices or for other reasons were defined as not receiving any therapies in the next simulation. Such methods were found in Markov model evaluations (3). Compliance not considered in the analysis. Lachaine et al. (16) assumed that patients remained on their medication continually for 5 years which was chosen as relatively short period to reduce uncertainty. Additionally, Abdall-Razak et al. (34) and Németh et al. (32) did not introduce the non-persistence rate due to a lack of relevant data.

**Table 5:** Summary of the methods used to integrate medication compliance.

**References**	**Expression**		**Roles in the model**	**Source of the data**				**Statistical methods**
	**Compliance**	**Persistence**		**Retrospective study**	**Clinical trials**	**Observational study**	**Review**	
Einarson et al. (15)	√	√	(a) Act as branches behind chance nodes, (b) influence the probabilities for events	√	√	√		Simple average
Lachaine et al. (16)	×	×						NA
Park et al. (17)	×	√	Lead to therapy changes		√			Kaplan-Meier discontinuation curves
Dilla et al. (18)	×	√	Lead to therapy changes		√			
Anh et al. (19)	√	×	Not specified				√	Simple average
Lubinga et al. (20)	×	√	Lead to relapse		√			NA
Druais et al. (21)	×	√	(a) Act as transition probabilities, (b) influence relapse of the disease		√			NA
Lin et al. (21)	×	√	Lead to change or discontinuation of the therapy				√	NA
Rajagopalan et al. (23)	×	√	Act as transition probabilities		√		√	Regression analysis, partial assumption
Einarson et al. (24)	√	√	(a) Act as branches behind chance nodes, (b) influence the probabilities of events	√	√	√		Partial assumption, simple average
Einarson et al. (25)	√	√	(a) Act as branches behind chance nodes, (b) influence the probabilities of events	√	√	√		Partial assumption, simple average
Barnes et al. (26)	Not specified							NA
Einarson et al. (27)	×	√	(a) Act as branches behind chance nodes, (b) influence the probabilities of events		√			NA
Einarson et al. (28)	×	√	(a) Lead to change or discontinuation of the therapy, (b) influence the relapse of the disease	√	√			NA
Wiwat et al. (29)	√	√	Influence relapse of the disease		√			NA
Nuhoho et al. (30)	√	√	(a) Act as branches behind chance nodes, (b) influence the probabilities of events	√				NA
Aigbogun et al. (31)	×	√	Lead to change or discontinuation of the therapy		√			Indirect comparison based on data from clinical trials
Németh et al. (32)	×	×						NA
Zhao et al. (33)	√	√	Classified different types of patients	√	√	√		Assumption
Abdall-Razak et al. (34)	×	×						NA
Dutina et al. (35)	Not specified							NA
Arteaga et al. (36)	×	√	Act as transition probabilities	√	√			NA
Yi et al. (37)	×	√	Act as transition probabilities					NA
Lin et al. (38)	√	√	Classified different types of patients	√	√	√		NA
Jin et al. (39)	√	√	Persistence rate acting as transition probabilities while non-compliance seen as reason for non-persistence	√			√	NA

*NA, not applicable*.

Various sources of reference for compliance or persistence were adopted in the evaluations, including retrospective studies, clinical trial studies, and naturalistic studies. Simple averaging was the major method adopted for multisource data. Most discontinuation rates were collected from clinical trials. Even though there might be differences between the definitions of compliance and persistence, most of the studies did not explain or discuss this issue. Considering the definition of compliance, data from real-world studies might be superior as no intervention has been implemented to influence the medication behaviors of patients.

#### Utility

According to the health states defined in the model, the most commonly used utilities in the studies were those for stable schizophrenia, relapse without hospitalization, relapse with hospitalization and the disutilities of EPS, weight gain, and diabetes. Utilities and references are summarized in Tables 6, 7. Utilities of the stable state, relapse without hospitalization and relapse with hospitalization ranged from 0.65 to 0.919, 0.485 to 0.762, and 0.27 to 0.604, respectively. Apart from EPS, weight gain and diabetes, the utilities or disutilities of hypertension, coronary heart disease, stroke, and hyperprolactinemia were also considered in some studies. Utilities from Lenert et al. (45) and Briggs et al. (46) were the most frequently referenced among the studies.

**Table 6:** Summary of the utilities for health states of schizophrenia.

**Study**	**Stable**	**Non-hospitalized relapse**	**Hospitalized relapse**	**References**
Einarson et al. (15)	0.89	0.659	0.49	(45–49)
Lachaine et al. (16)	0.75	NA	NA	(45)
Park et al. (17)	0.856	NA	−0.358	(46)
Dilla et al. (18)	0.77	—	−0.18	From SOHO data
Druais et al. (21)	0.919	0.762	0.604	(46)
Lin et al. (22)	0.8	NA	0.67	(45)
Rajagopalan et al. (23)	0.799	NA	0.67	(45, 46, 50)
Einarson et al. (24)	0.89	0.659	0.49	(45–49)
Einarson et al. (25)	0.89	0.659	0.49	(45–49)
Barnes et al. (26)	0.696	NA	NA	AMICUS trial
Einarson et al. (27)	0.7/0.65^*^	0.485/0.469^*^	0.27	(21, 51, 52)
Einarson et al. (28)	0.890/0.840/0.795/0.790^**^	0.690/0.665/0.643/0.640^**^	0.49	(21, 51, 52)
Wiwat et al. (29)	0.69	NA	0.58	(22)
Nuhoho et al. (30)	0.89	0.659	0.49	(45–49, 53)
Aigbogun et al. (31)	0.88	0.74	0.53	(45, 54)
Németh et al. (32)	Not reported	Not reported	Not reported	(39)
Zhao et al. (33)	0.88	0.74	0.53	(45), expert opinion
Abdall-Razak et al. (34)	0.799	0.67	0.67	(45)
Dutina et al. (35)	0.919	0.604	0.604	(46)
Arteaga et al. (36)	0.916/0.865^##^	−0.358	−0.358	(46, 51)
Yi et al. (37)	0.92	0.74^#^	0.60	(45, 46)
Lin et al. (38)	0.88/0.75/0.75^###^	0.74/0.63/0.63^###^	0.53/0.53/0.42^###^	(45), expert opinion
Jin et al. (39)	0.80	0.67	NA	(45)

*NA, not applicable*.

* *Data presented as utility for patients receiving injection therapy monthly/utility for patients receiving injection therapy every 3 months*.

** *Data presented as utility for patients receiving paliperidone 3-month injection/paliperidone 1-month injection/risperidone long-acting injection/haloperidol decanoate injection, oral olanzapine and clozapine*.

# *Data refer to acute phase*.

## *Data presented as utility for patients receiving paliperidone 3-month injection/paliperidone 1-month injection*.

### *Data presented as utility for full compliance, partial compliance and non-compliance patients*.

**Table 7:** Summary of the utilities of adverse events or complications.

**Study**	**EPS**	**Weight gain**	**Diabetes**	**Others**	**References**
Einarson et al. (15)	NA				
Lachaine et al. (16)	−0.074	−0.031	−0.06/−0.05^*^	Hypertension: −0.02 CHD: −0.07/−0.06^*^ stroke: −0.17/−0.18^*^	(45, 55)
Park et al. (17)	−0.256	NA	−0.151	Hyperprolactinemia: −0.089 CHD: −0.151	(46)
Dilla et al. (18)	−0.054	−0.003	NA	Sexual disfunction: −0.066	From SOHO data
Druais et al. (21)	−0.197	−0.094	−0.15		(46)
Lin et al. (22)	0.72^***^	0.77^***^	0.77^***^	myocardial infarction: 0.74^***^ stroke: 0.64^***^ poststroke: 0.70^***^	(45, 56, 57)
Rajagopalan et al. (23)	89%^**^	96%^**^	−0.15	NA	(45, 46, 50)
Einarson et al. (24)	NA				
Einarson et al. (25)	NA				
Barnes et al. (26)	NA	NA	NA	Adverse events-0.006	AMICUS trial
Einarson et al. (27)	NA	NA	NA	NA	NA
Einarson et al. (28)	NA	NA	NA	NA	NA
Wiwat et al. (29)	0.62^***^	0.66^***^	0.66^***^	Hyperprolactinemia: 0.62	(22, 58)
Nuhoho et al. (30)	NA				
Aigbogun et al. (31)	−0.099	−0.036	NA	Akathisia: −0.09 pathoglycemia: −0.067 dyslipidemia: −0.099 sedation: −0.084	(45, 59–61)
Németh et al. (32)	Not reported	Not reported	Not reported	Not reported	(62)
Zhao et al. (33)	88.8%^**^	95.9%^**^	NA	NA	(45)
Abdall-Razak et al. (34)	88.8%^**^	95.9%^**^	0.76^***^	Amputation: −0.109, non-fatal myocardial infarction: −0.129, non-fatal stroke: −0.181, heart failure: −0.108, ischemic heart disease: −0.132	(45, 50, 56, 63)
Dutina et al. (35)	−0.256	NA	NA	Metabolic syndrome: −0.132	(46)
Arteaga et al. (36)	−0.256	−0.089	−0.151	Prolactin-related syndrome: −0.089	(46)
Yi et al. (37)	0.72^***^	0.83^***^	NA	increased blood glucose level: 0.77^***^, liver function damage 0.75^***^, hyperprolactinemia: 0.82^***^	(45, 64)
Lin et al. (38)	88.8%^**^	95.9%^**^	88.8%^**^	hyperlipidemia/hyperprolactinemia: 88.8%^**^	(45)
Jin et al. (39)	−0.07	−0.03	−0.09	NA	(45, 56)

* *Data presented as utility for male/utility for female*.

** *Data presented as percentage reduction in utility for the presence of adverse effects*.

*** *Data presented as utility values*.

It was noteworthy that the utilities for the same disease states varied greatly. Possible reasons included varieties in the classification of the researched states, selection of the population, and different methods or instruments applied among referenced quality of life studies.

Definitions or classifications of the health states were usually developed based on a literature review (45, 46), expert opinions (45, 46) and interviews with patients or laypersons (46) rather than through a unified method. Such states are usually framed by several items or descriptions; however, few studies discussed the applicability for states in decision-analytical models.

Layperson, patients with schizophrenia and caregivers are common responders in research on health-related quality of life. The differentiation of responders could induce heterogeneity among the results. For example, Briggs et al. (46) discovered significant differences between laypersons and patients, especially for relapse and EPS states. Regardless of the doubt regarding the response ability of patients with schizophrenia, studies have demonstrated that schizophrenia patients in the stable stage were able to provide valid and reliable answers, indicating the necessity to include such a population (48, 53).

Due to the specialty of mental disease as well as the choice of population such as laypersons, caregivers, or psychiatrists, the majority of the methods generated utilities using the standard gambling or time trade off approaches. The EuroQol-5 dimensions (EQ-5D) questionnaire is preferred by The National Institute for Health and Care Excellence (NICE). However, the sensitivity of the EQ-5D index to capture both social and psychological well-being for patients with schizophrenia is still controversial (65).

### Results of the Economic Evaluations

The included studies compared the cost-effectiveness of commonly used SGAs (including long-acting injections). However, due to economic, political, cultural diversities among the different regions, the results of one economic evaluation may not be applicable beyond the defined setting (12). Thus, the results of the evaluation are not to be introduced here, but detailed information would be provided in the Supplementary Material 1. All of the studies adopted sensitivity analysis to verify the robustness of the base-case results. Notably, a change in the time horizon in different scenarios could lead to inverse results (17, 32). Accordingly, discussion about the time horizon may be required for schizophrenia therapy.

### Quality Assessment of the Included Studies

The widely used QHES and CHEERS lists economic evaluation checklists were applied for a quantitative and qualitative review.

**Figure 2:**
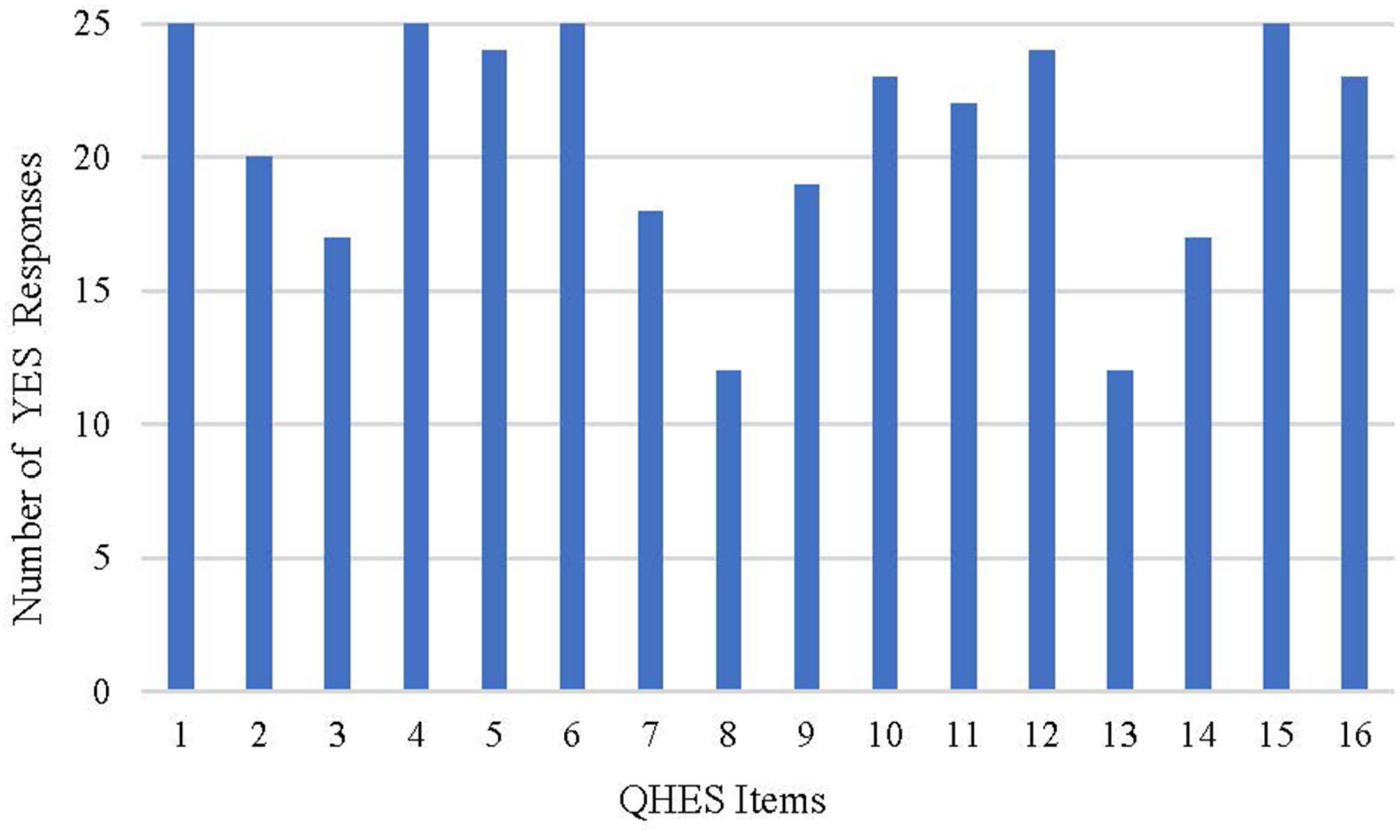
Results of the assessment with the QHES list.

Assessed with the QHES list, scores ranged from 60 to 93 for 25 studies, where 21 (84.0%) studies scored between 75 and 93, and 4 (16.0%) studies scored between 60 and 74, indicating the relatively high quality of the majority studies. As summarized in Table 8 and Figure 2, all of the studies met the requirements of item 1, item 4, item 6, and item 15 representing the descriptions of study objective, subgroup analysis, incremental analysis, and conclusion, respectively. However, no more than half of the studies met the requirements of item 8 (choice of appropriate time horizon and discount rate) and item 13 (statement and justification of the choice of model, assumptions, and limitations).

**Table 8:** Quality assessment of economic evaluations with the QHES checklist.

**Study**	**QHES items**																**Scores**
	**1**	**2**	**3**	**4**	**5**	**6**	**7**	**8**	**9**	**10**	**11**	**12**	**13**	**14**	**15**	**16**	
Einarson et al. (15)	√	×	×	√	√	√	√	×	√	√	√	√	√	√	√	√	81
Lachaine et al. (16)	√	√	√	√	√	√	√	√	√	√	√	√	×	√	√	√	93
Park et al. (17)	√	√	√	√	√	√	√	√	√	√	×	√	√	√	√	√	93
Dilla et al. (18)	√	√	√	√	√	√	×	√	√	√	×	√	√	√	√	×	85
Anh et al. (19)	√	√	×	√	√	√	√	√	√	√	√	√	×	×	√	×	76
Lubinga et al. (20)	√	√	√	√	√	√	√	√	√	√	√	√	×	×	√	√	87
Druais et al. (21)	√	√	√	√	√	√	√	×	√	√	√	√	√	√	√	√	93
Lin et al. (22)	√	√	√	√	√	√	√	√	×	√	√	√	×	√	√	√	85
Rajagopalan et al. (23)	√	√	×	√	√	√	√	√	√	√	√	√	√	×	√	√	86
Einarson et al. (24)	√	√	√	√	√	√	×	×	×	√	√	√	×	√	√	√	73
Einarson et al. (25)	√	√	√	√	√	√	×	×	√	√	√	√	×	√	√	√	81
Barnes et al. (26)	√	√	√	√	√	√	√	×	√	√	√	√	×	√	√	√	86
Einarson et al. (27)	√	√	√	√	√	√	√	×	√	√	√	√	×	√	√	√	86
Einarson et al. (28)	√	√	√	√	√	√	√	×	×	√	√	√	×	√	√	√	78
Wiwat et al. (29)	√	√	√	√	√	√	√	√	×	√	√	√	√	×	√	√	86
Nuhoho et al. (30)	√	√	×	√	√	√	√	×	×	√	√	√	√	√	√	√	77
Aigbogun et al. (31)	√	√	√	√	√	√	√	×	√	√	√	√	×	√	√	√	86
Németh et al. (32)	√	×	√	√	√	√	×	×	√	×	√	×	√	√	√	√	70
Zhao et al. (33)	√	√	×	√	√	√	√	×	√	√	×	√	√	√	√	√	78
Abdall-Razak et al. (34)	√	√	×	√	×	√	×	×	√	√	√	√	×	√	√	√	64
Dutina et al. (35)	√	√	×	√	√	√	×	√	×	√	√	√	×	×	√	√	60
Arteaga et al. (36)	√	×	√	√	√	√	√	√	√	√	√	√	√	√	√	√	96
Yi et al. (37)	√	√	√	√	√	√	√	√	√	√	√	√	×	×	√	√	87
Lin et al. (38)	√	×	√	√	√	√	×	×	√	√	√	√	√	×	√	√	78
Jin et al. (39)	√	×	×	√	√	√	√	√	√	√	√	√	√	×	√	√	82
Qualified studies	25/25	20/25	17/25	25/25	24/25	25/25	18/25	12/25	19/25	23/25	22/25	24/25	12/25	17/25	25/25	23/25	

**Figure 3:**
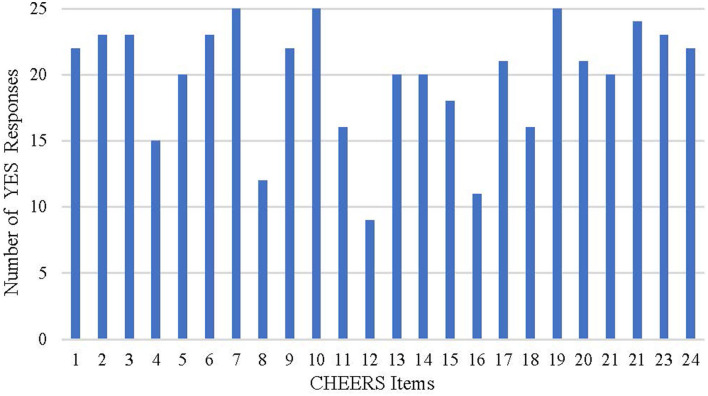
Results of the assessment with CHEERS checklist.

The quality reports evaluated with the CHEERS checklists were showed in Table 9 and Figure 3. The fulfilled items ranged from 16 to 23 for each study. More than half of the studies failed to meet the requirements for time horizon, preference-based outcome measurement, and assumptions. A considerable number of the studies selected a relatively short time horizon, which may be insufficient to capture necessary events. Most of the studies did not fully explain the reasons for time horizon selections. A large number of the studies chose utility values from the published literature, while some studies did not describe the methods used to elicit preference for outcomes. Studies seldom reported or explained the assumptions in single paragraph or table, adding to the difficulty identifying all assumptions underpinning the decision-analytical model. In addition, descriptions were for the parameters included, such as selection of population, measurement of effectiveness, and choice of model type.

**Table 9:** Quality assessment report of economic evaluations with the CHEERS checklist.

**Study**	**CHEERS items**																								**Fulfilled items**
	**1**	**2**	**3**	**4**	**5**	**6**	**7**	**8**	**9**	**10**	**11**	**12**	**13**	**14**	**15**	**16**	**17**	**18**	**19**	**20**	**21**	**22**	**23**	**24**	
Einarson et al. (15)	×	√	√	×	√	×	√	×	√	√	√	×	√	√	√	×	√	√	√	√	√	√	√	√	18/24
Lachaine et al. (16)	√	√	√	√	√	√	√	√	√	√	×	×	√	√	√	×	√	√	√	√	√	√	√	√	21/24
Park et al. (17)	√	√	√	×	√	√	√	√	√	√	√	×	√	√	×	√	√	√	√	√	√	√	√	√	21/24
Dilla et al. (18)	√	√	√	√	√	√	√	√	√	√	√	×	√	√	√	×	√	√	√	√	√	√	×	√	21/24
Anh et al. (19)	×	×	√	√	√	√	√	√	√	√	√	√	√	√	√	×	√	√	√	×	√	√	×	×	18/24
Lubinga et al. (20)	×	√	√	√	√	√	√	√	√	√	√	×	√	√	×	√	√	√	√	√	√	√	√	√	21/24
Druais et al. (21)	√	√	√	√	√	√	√	×	√	√	√	√	√	√	√	√	√	√	√	√	√	√	√	√	23/24
Lin et al. (22)	√	×	√	√	√	√	√	√	×	√	√	×	×	√	√	×	√	√	√	√	√	√	√	√	19/24
Rajagopalan et al. (23)	√	√	√	√	√	√	√	√	√	√	√	√	√	√	√	√	√	×	√	√	√	√	√	√	23/24
Einarson et al. (24)	√	√	√	√	√	√	√	×	√	√	√	×	×	√	√	×	√	×	√	×	×	√	√	×	16/24
Einarson et al. (25)	√	√	√	√	√	√	√	×	√	√	√	×	√	√	√	×	√	×	√	√	×	√	√	√	19/24
Barnes et al. (26)	√	√	√	×	√	√	√	×	√	√	√	√	×	√	×	×	×	×	√	×	×	√	√	√	15/24
Einarson et al. (27)	√	√	√	√	√	√	√	×	×	√	√	√	√	√	√	×	√	√	√	√	√	√	√	√	21/24
Einarson et al. (28)	√	√	√	×	√	√	√	×	√	√	√	√	×	√	×	×	√	×	√	√	√	√	√	√	18/24
Wiwat et al. (29)	√	√	√	√	√	×	√	√	√	√	×	×	√	×	√	√	√	√	√	√	√	√	√	×	19/24
Nuhoho et al. (30)	√	√	√	×	√	√	√	×	√	√	×	×	√	×	√	×	√	×	√	√	√	√	√	√	17/24
Aigbogun et al. (31)	√	√	√	√	√	√	√	×	√	√	√	√	√	√	×	√	√	√	√	√	√	√	√	√	22/24
Németh et al. (32)	√	√	×	√	×	√	√	×	√	√	×	√	√	√	×	√	√	√	√	√	√	√	√	√	19/24
Zhao et al. (33)	√	√	√	×	√	√	√	×	√	√	×	√	√	√	√	√	√	×	√	√	√	√	√	√	20/24
Abdall-Razak et al. (34)	√	√	√	×	√	√	√	×	√	√	×	×	√	√	×	×	×	×	√	×	√	√	√	√	15/24
Dutina et al. (35)	√	√	√	×	√	√	√	√	√	√	×	×	×	√	√	×	×	×	√	√	×	√	√	√	16/24
Arteaga et al. (36)	√	√	×	√	×	√	√	×	√	√	√	×	√	×	√	√	√	√	√	√	√	×	√	√	18/24
Yi et al. (37)	√	√	√	×	×	√	√	√	×	√	×	×	√	×	√	×	√	√	√	√	√	√	√	√	17/24
Lin et al. (38)	√	√	×	√	×	√	√	×	√	√	×	×	√	×	√	√	×	√	√	√	√	×	√	√	17/24
Jin et al. (39)	√	√	√	×	×	√	√	√	√	√	√	×	√	√	√	√	√	√	√	√	√	√	√	√	22/24
Qualified studies	22/25	23/25	23/25	15/25	20/25	23/25	25/25	12/25	22/25	25/25	16/21	9/25	20/25	20/25	18/25	11/25	21/25	16/25	25/25	21/25	20/25	24/25	23/25	22/25	

Unlike the CHEERS checklist acting as a recommendation of the report format, the QHES list was developed to appraise the quality of economic evaluation (13, 14). According to the assessment of the QHES and CHEERS lists, most of the studies could be identified as relatively high-quality analyses, but certain limitations existed regarding the report quality. The selection of an appropriate time horizon and description of assumptions are frequent limitations for the included studies or even for economic evaluations in other chronic diseases (66, 67).

To compare the quality among the included studies, model types, regions, and time horizons were used as the indicators to classify the studies. The average QHES score of Markov model studies was 84.46 (13 studies) which was higher than that of decision tree model studies (77.14, 7 studies). The average score of the studies applying microsimulation was 80.75. The average numbers of the items consistent with the CHEERS recommendations were 19.23, 17.71, and 20 for Markov model studies, decision tree model studies, and microsimulation model studies. As a result, the Markov model and microsimulation model rather than the decision tree model are more appropriate model types for the study of schizophrenia. The quality of studies among different regions was also slightly different. The average QHES scores of the studies from North America, Asia, and European countries were 90.67, 81.00, and 80.07, respectively. It should be noticed that certain discrepancies exist among the scores of the studies from European countries where the maximum and minimum scores were 96 and 64. The numbers of the items consistent with the CHEERS recommendations (21.3, 18.14, and 18.86, respectively) were similar. However, even though the studies from North America seemed to be of higher quality, the numbers of the studies from the three regions differed a lot (three studies from North America, 7 studies from Asia, and 14 studies from European countries) and this might introduce bias when assessing the qualities.

To analyze the quality differences of studies with different time horizons, we classified the studies into two categories: short-term studies, i.e., the time horizon was 1 year or less, and long-term studies, i.e., the time horizon was longer than 1 year. The averaged QHES scores of the short-term studies (78.2, 10 studies) were lower than that of the longer-term studies (84.3, 15 studies). However, the number of the items consistent with the CHEERS recommendations are similar (18.3 for short-term studies vs. 19.53 for long-term studies). As mentioned above, the QHES list was developed to appraise the quality of economic evaluation while the CHEERS checklist was developed to recommend the report format. It can be inferred from the description of the time horizon from the two lists that the QHES list (Did the analytic horizon allow time for all relevant and important outcomes?) was more subjective and focused on the relationship between the item assessed and model outcomes. While the CHEERS checklist [State the time horizon(s) over which costs and consequences are being evaluated and say why appropriate] highlighted the fact that the statement of relevant aspect was provided and with no requirement to judge the appropriateness for the evaluation. Thus, attention should be paid when choosing checklists and interpreting the results.

## Discussion

### Purpose of Systematic Review of Economic Evaluations

With the increasing number of publications on health economic studies in recent years, systematic reviews in this filed have caught the attention of decision makers as useful tools to generate evidence (68). However, concerns have been voiced regarding whether cost-effectiveness findings can be transferred from one setting to another (12). Anderson et al. discussed the generalizability of the results of health economic studies and concluded that differences in methods, context, intervention costs, and effects contributed to the limitations on evidence synthesis (12). Gomersall et al. also discussed the debate over futility vs. utility of systematic reviews of economic evidence, emphasizing the inability to compare resources between countries and the differences in context and population (69). Pigone et al. also pointed out the challenges in the presentation of economic systematic reviews due to the large amounts of both synthesized data and generated results (70).

Despite challenges in generating results of economic evaluations via systematic reviews, it was suggested that systematic reviews should focus on methods of model development, sources of both efficacy and utility data, and resources used for specific diseases. It might be valuable for researchers and decision makers to identify the differences among studies (69, 70). For model-based economic evaluations, in particular, model structure selection was recommended based on the summarized existing studies, which could provide relatively comprehensive consideration for the necessary model states and link clinical practice and hypothetical model transitions. Notably, compared with differences in national contexts, it was found that variability in published economic evaluations was related more to the variety of study (71), confirming the importance of summarizing the model methods applied to existing studies. Thus, this systematic review focuses on the methods and study quality of the 25 eligible studies.

### Main Findings

Among the included studies, most compared the cost-effectiveness of SGAs or long-acting injections. Fifteen studies considered the treatment sequences in model development, which could enlighten the future model development.

Despite the burden and productivity loss due to schizophrenia, few studies chose the societal perspective covering the indirect costs. Due to the early onset of schizophrenia, the average age of these patients is younger than that of patient with other chronic diseases. It would be necessary to include indirect costs in the evaluation. A retrospective study in the US concluded that indirect and non-health care costs were strong contributors and could be more than 70% of the total burden (72). A systematic review of the indirect costs of schizophrenia in Europe found that the average proportion of indirect costs of total disease expenditure was 44% (73). In China, the per case per annum indirect costs of schizophrenia were approximately US$1723.4 in 2013, accounting for 66.6% of the total costs (74). Therefore, a societal perspective covering both direct and indirect costs is preferable.

Description of the treatment sequences from most of the included studies could improve the model design and reflect the clinical prescription especially for chronic diseases or patients with high rate of therapy change. However, there remains challenges considering treatment sequences in economic evaluation. Medication treatment may vary among individuals due to the genotypes, metabolism, comorbidities, adverse events and so on (75). Also, the followed medication therapy should be impact by the previous medication choices. Thus, it may be questioned to apply a uniform sequency for patients especially in the cohort model. Cost and effectiveness data for the multiple drugs and the analytical methods are other challenges for treatment sequences. Even though choice of single drug based on market share or expert opinions and weighted or unweighted averaged data from multiple drugs are common methods in studies, the appropriateness requires further discussion.

According to the summary of the models applied, the Markov model was the most frequently used and treatment sequences, relapse, remission, and adverse events were the important health state elements in model development. The time horizons varied from 5 years to lifetime for the Markov models. While for the decision tree models, a 1 year time horizon was preferred. Due to the uncertainty in the time frame of treatment, it is recommended the impact of the time horizon be explored in a sensitivity analysis. Adverse events such as EPS, weight gain, and diabetes should be considered in the models for schizophrenia since these factors have a recognized influence on the treatment effect. Also, consideration of different types of AEs should be properly defined to estimate the impact on health outcomes and costs in the economic evaluation.

Based on the criteria studies, compliance and persistence were not clearly classified, thus definition is recommended for economic evaluations since it might determine the choice of appropriate data source. When integrating compliance or persistence, data are required on both health outcomes and costs for patients who are non-compliant or discontinued treatment (76). Evidence for compliance or persistence could be collected from retrospective studies, clinical trials, observational studies, or reviews.

Utility values, derived from the literature may contribute to the heterogeneity among results when they are applied to the same health states in different studies. Differences in the classifications of health states, survey responders, elicitation methods, and regions were the main factors influencing the utility values. Thus, it is recommended that researchers choose proper sources based on the factors above, as well as the publication year or update of methods.

Certain limitations to study quality have been identified, such as the description of appropriate time horizon selection, discount rate, statement, and justification of the choice of model type, assumptions and limitations to the evaluations. For the reporting quality of the studies, time horizon, preference-based outcome measurement, and assumptions were the major missing parts. To improve both the quality of the study and the quality of the report, it is suggested that researchers conduct the evaluation and generate the manuscript under the respective guidance and checklists.

Though there exist studies reviewed the economic evaluation of treatment for schizophrenia (8–10, 77), few fully discussed the treatment sequences, AEs, compliance and persistence of the included studies. Thus, this review provides more comprehensive and detailed information of modeling methodology for economic evaluation.

### Limitations

There remain some limitations of this review. First, the review only included studies published after 2014 and does not represent the economic methods used in earlier years. Studies published recently may be more valuable for analysis, considering the relatively high quality, most recent treatment options and updated clinical evidence. Second, this review only included model-based economic evaluations. Even though trial-based economic evaluations for schizophrenia are also important evidence, this study aims to generate summaries and suggestions for model methodology rather than synthesizing economic evidence. In addition, trial-based studies may not provide long-term clinical outcomes and source consumption, especially for chronic diseases.

### Suggestions

Based on the results of this review, it is suggested that future research focus on methods to integrate compliance or persistence data for chronic diseases. Due to the diverse utilities cited in the models, characteristics of study groups and measuring approach of preference-based health outcomes from the health-related quality of life research should be explained to provide appropriate options for the studies. Publications of economic evaluations should be designed and reported according to applicable gelines and checklists to improve study quality and provide both scientific and valuable evidence for decision makers. Future research could pay more attention to the economic evaluation of long-acting injection antipsychotics.

## Data Availability Statement

The original contributions presented in the study are included in the article/Supplementary Material, further inquiries can be directed to the corresponding author.

## Author Contributions

LW and HL contributed to the study design, analysis, and writing. FS, XG, and HX contributed to the review work. JL contributed to the manuscript revise. All authors contributed to the article and approved the submitted version.

## Funding

This study received funding from Sumitomo Pharma (Suzhou) Co., Ltd.

## Conflict of Interest

This study received funding from Sumitomo Pharma (Suzhou) Co., Ltd. The funder had the following involvement with the study: decision to published. JL was employed by company Sumitomo Pharma. The remaining authors declare that the research was conducted in the absence of any commercial or financial relationships that could be construed as a potential conflict of interest.

## Publisher's Note

All claims expressed in this article are solely those of the authors and do not necessarily represent those of their affiliated organizations, or those of the publisher, the editors and the reviewers. Any product that may be evaluated in this article, or claim that may be made by its manufacturer, is not guaranteed or endorsed by the publisher.
