# Supplementary material for: A Systematic Review of Methods and Study Quality of Economic Evaluations for the Treatment of Schizophrenia
Source: Front Public Health. 2021 Oct 20;9:689123. doi: 10.3389/fpubh.2021.689123 (PMC8564012; doi:10.3389/fpubh.2021.689123)
Supplement: Supplementary file 1 [file Data_Sheet_1.docx]

Supplementary Material

# Searched Details from Databases

## Supplementary Table 1. Searched details from Pubmed

**Table 1. Searched Details from Pubmed**

| Item | Search query | Results |
| --- | --- | --- |
| 1 | schizophreni*[TIAB] | 130175 |
| 2 | pharmacoeconomics[TIAB] | 1629 |
| 3 | cost effectiveness[TIAB] | 63419 |
| 4 | cost utility[TIAB] | 5134 |
| 5 | economic evaluation[TIAB] | 10579 |
| 6 | 2 OR 3 OR 4 OR 5 | 71323 |
| 7 | 2014/01/01:2020/12/31[DP] | 8397783 |
| 8 | 1 AND 6 AND 7 | 202 |

TIAB: title or abstract; DP: data of publication

## Supplementary Table 2. Searched details from Web of Science

**Table 2. Searched Details from Web of Science**

| Item | Search query | Results |
| --- | --- | --- |
| 1 | TI=schizophreni* | 119,441 |
| 2 | AB=schizophreni* | 112,508 |
| 3 | 1 OR 2 | 169,112 |
| 4 | TI=pharmacoeconomic | 2,244 |
| 5 | TI=cost effectiveness | 37,453 |
| 6 | TI=cost utility | 3,572 |
| 7 | TI=economic evaluation | 10,906 |
| 8 | 4 OR 5 OR 6 OR 7 | 53,212 |
| 9 | AB=pharmacoeconomic | 2,702 |
| 10 | AB=cost effectiveness | 121,612 |
| 11 | AB=cost utility | 29,972 |
| 12 | AB=economic evaluation | 41,393 |
| 13 | 9 OR 10 OR 11 OR 12 | 178,424 |
| 14 | 13 OR 8 | 204,665 |
| 15 | 3 AND 14 AND PY=(2014-2020) | 377 |

TI: title; AB: abstract; PY: publication year

## Supplementary Table 3. Searched Details from EBSCOhost, Academic Search Ultimate

**Table 3. Searched Details from EBSCOhost, Academic Search Ultimate**

| Item | Search query | Results |
| --- | --- | --- |
| 1 | TI schizophreni* | 45,464 |
| 2 | AB schizophreni* | 68,032 |
| 3 | 1 OR 2 | 78,204 |
| 4 | TI pharmacoeconomic | 1,177 |
| 5 | TI cost effectiveness | 15,682 |
| 6 | TI cost utility | 1,366 |
| 7 | TI economic evaluation | 4,160 |
| 8 | 4 OR 5 OR 6 OR 7 | 22,074 |
| 9 | AB pharmacoeconomic | 2,681 |
| 10 | AB cost effectiveness | 39,952 |
| 11 | AB cost utility | 2,529 |
| 12 | AB economic evaluation | 6,839 |
| 13 | 9 OR 10 OR 11 OR 12 | 47,185 |
| 14 | 13 OR 8 | 56,041 |
| 15 | 3 AND 14 | 383 |
| 16 | 3 AND 14 with data of publishment limitation: 20140101-20201231 | 113 |

TI: title; AB: abstract

## Supplementary Table 4. Searched Details from ScienceDirect

**Table 4. Searched Details from ScienceDirect**

| Search field | Search query | Results |
| --- | --- | --- |
| title, abstract or author-specified keywords | (schizophrenia OR schizophrenic) AND (((pharmacoeconomic OR economic evaluation) OR cost effectiveness) OR cost utility) | 108 |
| year range | 2014-2020 |  |

## Supplementary Table 5. Searched Details from Cochrane Library

**Table 5. Searched Details from Cochrane Library**

| Search field | Search query | Results |
| --- | --- | --- |
| title | (schizophrenia OR schizophrenic) AND (pharmacoeconomic OR economic evaluation OR cost effectiveness OR cost utility) | cochrane reviews: 0; trials: 22 |
| year(s) | 2014-2020 |  |
| abstract | (schizophrenia OR schizophrenic) AND (pharmacoeconomic OR economic evaluation OR cost effectiveness OR cost utility) | cochrane reviews:48; trials: 216 |
| year range | 2014-2020 |  |
| Total | | 286 |

# Summaries of Base Case and Sensitivity Analysis Results

## Supplementary Table 6. Summary of the Base Case Results and Conclusions

**Table 6. Summary of the Base Case Results and Conclusions**

| Study | WTP | Base Case Results | Conclusions |
| --- | --- | --- | --- |
| Einarson 2014 | not specify | The 1st-line paliperidone LAI therapy followed by the 2nd-line olanzapine LAI therapy and the 3rd-line clozapine therapy was the dominant strategy with reduced cost and additional QALYs. | The 1st-line paliperidone followed by the 2nd-line olanzapine LAI and the 3rd-line clozapine strategy was the most cost-effective strategy in Sweden. |
| Lachaine 2014 | $50,000/QALY | Asenapine was a dominant strategy compared with olanzapine with reduced cost and additional QALYs. | Asenapine was a cost-effective strategy compared with most of the atypical antipsychotics frequently used in Canada. |
| Park 2014 | $50,000/QALY, $100,000/QALY | 1. The ICER was $542,500/QALY for the 1st-line ziprasidone followed by the 2nd-line quetiapine strategy compared with the 1st-line ziprazidone followed by the 2nd-line risperidone strategy. 2. The ICER was $5,200/QALY for the 1st-line ziprasidone followed by the 2nd-line risperidone strategy compared with the 1st-line risperidone followed by the 2nd-line ziprazidone strategy. | The 1st-line ziprasidone followed by the 2nd-line quetiapine strategy was the most cost-effective strategy in United States. |
| Dilla 2014 | € 30,000/QALY | Compared with risperidone LAI, olanzapine LAI was a dominant strategy with reduced cost and additional QALYs. | Olanzapine-LAI was a dominant strategy compared with risperidone-LAI in Spain. |
| Anh 2015 | $2,388/DALY (1-time per capital GDP) | 1. Compared with the do-nothing treatment, all of the selected drug interventions alone or in combination, except for clozapine, were cost-saving. 2. Intervention using clozapine were estimated to be very cost-effective with the ICER of $932/DALY averted.3. Compared with other typical antipsychotics, risperidone, olanzapine, and clozapine were more cost-effective. | Compared with the do-nothing treatment, other treatment strategies included in the study were more cost-effective in Vietnam. |
| Lubinga 2015 | unspecified | 1.Compared with haloperidol and quetiapine, risperidone was a dominant strategy. 2. The ICER was $5868/DALY averted for olanzapine compared with risperidone . | Risperidone was the most cost-effective strategy in Uganda. |
| Druais 2016 | € 30,000/QALY | 1.The ICER was €2411/QALY for paliperidone LAI compared with oral antipsychotics. 2. The ICER of risperidone LAI compared with paliperidone LAI was €47,70018/QALY. 3. Compared with aripiprazole LAI, olanzapine LAI and haloperidol LAI, lurasidone was a dominant strategy with reduced costs and additional QALYs. | Paliperidone LAI was the most cost-effective strategy in France. |
| Lin 2016 | SGD 70,000/QALY (1-time GDP) | Compared with other 10 oral antipsychotics, olanzapine was the least costly and most effective strategy. | Olanzapine was the most cost-effective strategy in Singapore. |
| Rajagopalan 2016 | £20000/QALY | Compared with aripiprazole, lurasidone was a dominant strategy with reduced costs and more effectiveness. | Lurasidone was a cost-effective strategy, especially in patients with schizophrenia at risk of weight gain and metabolic disease in Scotland and Wales. |
| Einarson 2016 | €24,800/QALY | Compared with other strategies, paliperidone LAI was a dominant strategy with reduced cost and more effectiveness. | Paliperidone LAI was a cost-effective strategy for chronic recurrent schizophrenia in Finland. |
| Einarson 2016 | €30,000/QALY €24,800/QALY | 1. The ICERs of Paliperidone LAI Compared with oral olanzapine were €14,247/QALY, €1973/relapse averted and €2697/hospitalization averted. 2. Compared with haloperidol LAI and risperidone LAI, paliperidone LAI was a dominant strategy with reduced cost and additional QALYs. | Haloperidol LAI, oral olanzapine and paliperidone LAI were more cost-effective compared with risperidone LAI in Portugal. |
| Barnes 2017 | £15,000/QALY | Amisulpride augmentation had the potential to be cost-effective in the short term with net saving between £329 and £2011, and possibly in the long-term. | Amisulpride–clozapine combination was a cost-effective strategy in United Kingdom. |
| Einarson 2017 | € 30,000/QALY | Compared with PP1M, PP3M was a dominant strategy in both cost-effective and cost-utility analyses. | PP3M was more cost-effective compared with PP1M in Spain. |
| Einarson 2017 | not specify | Compared with PP1M, haloperidol LAT, risperidone LAI and oral olanzapine, PP3M was a dominant strategy with reduced cost, additional QALYs and lower relapse. | PP3M was the most cost-effective strategy in Netherlands. |
| Thavornwattanayong 2018 | 160,000 Baht/QALY (1.2-time per capital GDP） | Compared with risperidone, aripiprazole was a dominant strategy with reduced cost and more effectiveness. | Aripiprazole was a cost-effective strategy in Thailand. |
| Nuhoho 2018 | $38,000/QALY | Compared with other oral antipsychotics, PP1M was a dominant strategy with reduced cost and more effectiveness. | PP1M was more cost-effective compared with other oral antipsychotics in United Arab Emirates. |
| Aigbogun 2018 | $30,000/QALY | Brexpiprazole was the least costly and most effective strategy of all strategies. | Brexpiprazole was more cost-effective compared with lurasidone and cariprazine in United States. |
| Németh MSc 2019 | € 34,764/QALY | The ICER was € 28,897/QALY for cariprazine compared with risperidone. | Cariprazine was more cost-effective compared with risperidone in Hungary. |
| Zhao 2019 | $25,772.67/QALY | 1. The ICERs were $ 2,791.96/relapse averted and $16798.39/QALY for olanzapine ODT compared with olanzapine SOT. 2. Olanzapine ODT was dominant over aripiprazole ODT more cost-effective compared with risperidone-LAI. with reduced cost and additional QALYs. | Olanzapine ODT was more cost-effective compared with olanzapine SOT. Olanzapine SOT was more cost-effective compared with aripiprazole SOT in China. |
| Abdall-Razak 2019 | £20,000/QALY, £30,000/QALY | The ICER was £1,0941/QALY for paliperidone compared with amisulpride. | Paliperidone is cost-effective compared with amisulpride in United Kingdom. |
| Dutina 2019 | RSD 70,462/QALY (1-time per capital GDP) | The ICER was 131,417 RSD/QALY for aripiprazole compared with olanzapine. | Olanzapine is cost-effective compared with aripiprazole in Serbian. |
| Duarte 2019 | $5,000/QALY | Compared with PP1M, PP3M was a dominant strategy with reduced cost and additional QALYs. | PP3M is cost-effective compared with PP1M in France. |
| Yi 2020 | $9322/QALY (1-time per capital GDP) | Compared with olanzapine, amisulpride was a dominant strategy with reduced cost and additional QALYs. | Amisulpride is likely to be a cost-effective option in China |
| Lin 2020 | $28,112/QALY (3-time per capital GDP) | 1. Compared with aripiprazole SOT, aripiprazole ODT was dominant option with reduced cost and additional cost. 2. Compared with olanzapine SOT, aripiprazole SOT was dominant option with reduced cost and additional cost. | The aripiprazole ODT is cost-effective compared with both aripiprazole SOT and olanzapine SOT in China. |
| Jin 2020 | £20,000/QALY | 1. When compared 1st-line oral antipsychotic medication for individuals with FEP: amisulpride was dominant over other options followed by risperidone and olanzapine. 2. When evaluated family intervention for individuals with FEP, antipsychotics plus family intervention dominated both antipsychotics alone and family intervention alone. 3. When compared 1st-line oral antipsychotics for individual with TRS, clozapine dominates other antipsychotics. | The current schizophrenia service configuration is not optimal and more cost-effective intervention should be applied in United Kingdom. |

LAI: long-acting-injection, ODT: orally disintegrating antipsychotic tablets, SOT: standard oral tablets, PP3M: paliperidone administered every 3 months, PP1M: paliperidone administered every month, QALY: quality-adjusted life year, DALY: disability-adjusted life year, ICER: incremental cost-effectiveness ratio SGD: Singapore dollar, RSD: Serbian dinar, FEP: first-episode psychosis, TRS: treatment-resistant schizophrenia, GDP: gross domestic product, WTP: willingness-to-pay

## Supplementary Table 7. Summary of the Sensitivity Analysis

**Table 7. Summary of the Sensitivity Analysis**

| Study | Results of Deterministic Sensitivity Aanalysis | Results of Scenario Analysis | Results of Probabilistic Sensitivity Analysis |
| --- | --- | --- | --- |
| Einarson 2014 | Results of the base case analysis were sensitive to adherence and the rates of hospitalization. | NA | Compared with the 1st-line olanzapine LAI followed by the 2nd-line paliperidone LAI and the 3rd-line clozapine, the 1st-line risperidone LAI followed by the 2nd-line haloperidol LAI and the 3rd-line Clozapine, the 1st-line haloperidol LAI followed by the 2nd-line oral olanzapine and the 3rd-line clozapine, the 1st-line oral olanzapine followed by the 2nd-line haloperidol LAI, the probabilities that the 1st-line paliperidone LAI followed by the 2nd-line olanzapine LAI and the 3rd-line clozapine strategy was more cost-effective were 59.4%, 65.8%, 94.0% and 95.5%, respectively. |
| Lachaine 2014 | Results of the base case analysis were stable. | Asenapine was still the dominant treatment in the 10-year scenario analysis. | Compared with olanzapine, the probability that asenapine was cost-effective was 100%. |
| Park 2014 | Results of the base case analysis were sensitive to the Cost of CHD, relapse rate, cost of risperidone, diabetes, and the probability of developing MS with risperidone. | 1. In 18-month and 5-year scenario analysis, risperidone was more cost-effective as first-line therapy. 2. Ziprasidone was more cost-effective as first-line therapy when study horizon exceeding 10 years. | 1. Compared with the risperidone- strategy, the probabilities that ZSD-RSP strategy was cost-effective were 88.82% and 89.80%. (The threshold of WTP were $50,000/QALY and $100,000/QALY, respectively.) 2. Compared with the ziprasidone - quetiapine strategy, the probabilities that ziprasidone - risperidone strategy was cost-effective were 99.96% and 98.82%. (The threshold of WTP were $50,000/QALY and $100,000/QALY, respectively.) |
| Dilla 2014 | NA | Olanzapine LAI was still the dominant treatment in the 5-year scenario analysis. | 1. Compared with risperidone LAI, the probabilities that olanzapine LAI was cost-effective were 84%, 80% and 72%. (with WTP thresholds of €100,000/QALY, € 75,000/QALY and €30,000/QALY, respectively.) 2. Results of base case analysis were stable. |
| Anh 2015 | NA | NA | 1. The scatter plot showed that risperidone and olanzapine kept to be the dominant strategies. 2. Results of base case analysis were stable. |
| Lubinga 2015 | 1. The residual and acute state disability weights had the largest impact on DALYs averted, followed by the probabilities of discontinuation. 2. Results of the base case analysis were stable. | Results of base case analysis were stable in the scenarios analysis except for the discounting scenario. | 1.The probabilities that risperidone was cost-effective were 85% and 74%. (with WTP threshold being 1-time GDP and 3-time GDP, respectively.) 2. Results of base case analysis were stable. |
| Druais 2016 | Results of base case analysis were sensitive to the rates of treatment interruption and relapse due to lack of efficacy. | Paliperidone was still an economic treatment based on the scenario of literature-sourced costs, longer hospital stays, changed dose of paliperidone and adjusted utility values. | 1. Paliperidone was the most cost-effective treatment. 2. Results of base case analysis were stable. |
| Lin 2016 | 1. The cost of treatment and the rates of relapse were the most important factors. 2. Results of the base case analysis were stable to other factors. | NA | 1.The probability that olanzapine was the most economic treatment was 75%. (with WTP threshold of SGD 50,000/QALY.)  2. Results of base case analysis were stable. |
| Rajagopalan 2016 | The rates of relapse were the most important influencing factor, which might change the results of the base case analysis. | Lurasidone was the most cost-effective treatment in scenario analysis. | 1. The probability that lurasidone was a cost-effective strategy was 75%. (with WTP threshold of £20,000/QALY.) 2. Results of base case analysis were stable. |
| Einarson 2016 | Results of the base case analysis were sensitive to changeable rates of adherence, dropout, relapse, and drug prices. | NA | Compared with aripiprazole LAI, risperidone LAI and olanzapine LAI, the probability that paliperidone LAI was the dominant strategy were 75.8%, 83.1% and 95.7%, respectively. (with WTP threshold of €24,800/QALY) |
| Einarson 2016 | Results of the base case analysis were stable when parameters changed. | NA | Compared with oral olanzapine, the probability that paliperidone was more cost-effective was more than 99%. (with WTP threshold of €24,800/QALY or €30,000/QALY) |
| Barnes 2017 | Results of the base case analysis were insensitive to parameter changes and were relatively stable. | NA | The probability that amisulpride augmentation was a cost-effective strategy varied between 54% and 59%, |
| Einarson 2017 | Results of base case analysis were insensitive to parameter changes and were relatively stable. | 1.PP3M remained to be a dominant strategy despite of changeable rates, such as hospitalization. 2. In the worst case scenario (with PP3M maximum dose), PP3M remained cost-effective with an ICER of €16,752/QALY. 3.PP3M kept dominant in the scenario of drug prices change, longer hospital stays and adjusted utility values. | Compared with PP1M, the probability that PP3M was a dominant strategy was 46.9%, the probability that PP3M was cost-effective was 77.8%. (with WTP threshold of €30,000/QALY.） |
| Einarson 2017 | Results of the base case analysis were insensitive to parameter changes and were relatively stable. | PP3M remained to be a dominant strategy despite of the change of drugs doses, dropout rates and alternative utility scores. | With DRGs cost or microcosting, the probability that PP3M was a dominant strategy was 85.0% and 84.6%, the probability that PP3M was cost-effective was 85.8% and 99.9%, respectively. |
| Thavornwattanayong 2018 | 1. The price of aripiprazole was the most influencing factor. 2. Results of base case analysis were insensitive to other factors. | NA | 1. The probability that aripiprazole was a cost-effective strategy was 97.5%. (with WTP threshold of 160,000 baht/QALY)  2. Results of base case analysis were stable. |
| Nuhoho 2018 | 1. The price of paliperidone was the most influencing factor.  2. Results of the base case analysis were stable to other factors. | NA | Compared with other oral antipsychotics, probabilities that paliperidone LAI was a dominant strategy or more cost-effective were 96% and 99.998%, respectively. (with WTP threshold of $38,000) |
| Aigbogun 2018 | Results of the base case analysis were sensitive to the rates of relapse and daily cost of brexpiprazole. | 1.Brexpiprazole kept to be dominant option in the scenario of changing the incidence of adverse events. 2. Brexpiprazole was sill the dominant option in using retail price. | Brexpiprazole was consistently a cost-effective treatment. (with WTP threshold of $30,000) |
| Németh MSc 2019 | 1.The change of utility values could change the results of the base case analysis. 2. Results of the base case analysis were stable to other factors. | 1. Results of basic analysis changed in the 1-year time horizon scenario. 2. Results of base case analysis were stable in other scenario analyses. | The probability that cariprazine was more cost-effective was 70%. (with WTP threshold of €34,764/QALY.) |
| Zhao 2019 | 1. The adherence of olanzapine ODT was the main influencing factor, which could change the conclusion. 2. Results of the basic analysis were stable to other factors. | NA | Compared with olanzapine SOT and aripiprazole, the probability that olanzapine ODT was more cost-effective was 84.4%. (with WTP threshold of $25,773/QALY.) |
| Abdall-Razak 2019 | Change of the probability of EPS could reverse the base case results. | 1. In the change of utility scenario, paliperidone remained to be cost-effective strategy. 2. In the higher cost of relapse scenario, paliperidone dominated amisulpride. 3. In the high costs of weight gain management scenario, Paliperidone remained to be cost-effective with increased ICER. | It would be reasonable to argue that paliperidone shoule be preferred to amisulpride as the ICER (£10,941/QALY) were lower than the National Health System threshold values. (with WTP threshold of £20,000 - £30,000) |
| Dutina 2019 | Results of the base case analysis were insensitive to parameter changes and were relatively stable. | NA | Compared with olanzapine the probability that aripiprazole was cost-effective was only 13% when WTP reached 9-time GDP per capital (with WTP threshold is 1-time GDP per capital) |
| Duarte 2019 | Changes in rates of relapse, AEs rates and discontinuation rate for PP3M were the important factors. Results of the base case were generally stable. | 1. In the high relapse rate scenario, saving effect of PP3M would increase.  2. In the reduced discontinuation rate for PP3M scenario, PP3M could generated higher increase in QALY compared with the base case. | Compared with PP1M, PP3M would have 99% probability of being cost-effective. (with WTP threshold of €5,000/QALY) |
| Yi 2020 | 1.Unit cost of olanzapine was the most sensitive variable. 2. The results of base case were stable to other variables. | 1. In the 5-year horizon scenario, amisulpride remained dominant over olanzapine. 2. In the 20-year-old starting age scenario, amisulpride remained to be the dominant option. | Compared with olanzapine, the likelihood of amisulpride being considered cost-effective was 99.8%. (with WTP threshold of $9,322) |
| Lin 2020 | 1. The model was most sensitive to changes in adherence to aripiprazole ODT for both comparisons. 2. adherence to aripiprazole SOT, adherence to olanzapine SOT and drug cost were also important variables. | NA | Compared with aripiprazole SOT, the likelihood of aripiprazole ODT being cost-effective was 99.2%. (with WTP threshold of $28,112) |
| Jin 2020 | 1.In the family intervention for individuals with FEP topics, the results were robust to other variables but changes in choice of 1st-line antipsychotics, effectiveness of family intervention and number of family intervention sessions provided. 2. Other topics are robust to the changes of variables. | Results of base case analysis were stable when changes were made to the structural assumptions of the model. | 1. In the 1st-line oral antipsychotics for individuals with FEP topic, the probabilities of amisulpride, risperidone and olanzapine being cost-effective are 39%, 30% and 17%, respectively under both £20,000 and the £30,000 thresholds. 2. In the family intervention for individuals with FEP topic, the probability of antipsychotics plus family intervention being cost-effective is 58% and 62% under the £20,000 and the £30,000 thresholds. 3. In the 1st-line oral antipsychotics for individuals with TRS topic, the probability of clozapine being cost-effective is 81% under both the £20,000 and the £30,000 thresholds. |

LAI: long-acting-injection, ODT: orally disintegrating antipsychotic tablets, SOT: standard oral tablets, PP3M: paliperidone administered every 3 months, PP1M: paliperidone administered every month, QALY: quality-adjusted life year, DALY: disability-adjusted life year, ICER: incremental cost-effectiveness ratio SGD: Singapore dollar, RSD: Serbian dinar, FEP: first-episode psychosis, TRS: treatment-resistant schizophrenia, CHD: cardiovascular heart disease, MS: metabolic syndrome, EPS: extrapyramidal symptoms, AEs: adverse events, GDP: gross domestic product, WTP: willingness-to-pay, NA: not applicable
